# The critical role of endothelial cell in the toxicity associated with chimeric antigen receptor T cell therapy and intervention strategies

**DOI:** 10.1007/s00277-024-05640-z

**Published:** 2024-02-08

**Authors:** Qi Zhang, Xiaojian Zhu, Yi Xiao

**Affiliations:** Department of Hematology, Tongji Hospital, Tongji Medical College, Huazhong University of Science and Technology, Wuhan, Hubei China

**Keywords:** Chimeric antigen receptor-T cell therapy, Endothelial cells, Cytokine release syndrome, Immune effector cell-associated neurotoxic syndrome, Angiotensin-1, Angiotensin-2

## Abstract

Chimeric antigen receptor (CAR)-T cell therapy has shown promising results in patients with hematological malignancies. However, many patients still have poor prognoses or even fatal outcomes due to the life-threatening toxicities associated with the therapy. Moreover, even after improving the known influencing factors (such as number or type of CAR-T infusion) related to CAR-T cell infusion, the results remain unsatisfactory. In recent years, it has been found that endothelial cells (ECs), which are key components of the organization, play a crucial role in various aspects of immune system activation and inflammatory response. The levels of typical markers of endothelial activation positively correlated with the severity of cytokine release syndrome (CRS) and immune effector cell-associated neurotoxic syndrome (ICANS), suggesting that ECs are important targets for intervention and toxicity prevention. This review focuses on the critical role of ECs in CRS and ICANS and the intervention strategies adopted.

## Introduction

Chimeric antigen receptor (CAR)-T cell therapy is a rapidly advancing tumor immunotherapy approach that has been widely used and shown to be effective in the treatment of hematologic malignancies, including acute lymphoblastic leukemia [1, 2], non-Hodgkin’s lymphoma [3–5], and multiple myeloma [6, 7]. A certain amount of cytokine release is a marker of efficacy; however, excessive cytokine release leads to treatment-related toxicity. Cytokine release syndrome (CRS) and immune effector cell-associated neurotoxic syndrome (ICANS), which most treatment-related deaths are attributed to, are the most common and severe toxicities that require early intervention and standardized management [8–10].

The development and severity of CRS cannot be effectively predicted and attenuated by adjusting the relevant factors affecting its growth and severity, such as the number and type of CAR-T infusions [11, 12]. Simultaneously, treatment with interleukin (IL)-6 inhibitors is effective only during the early stages of CRS. This may cause severe ICANS due to binding to IL-6R, which allows high levels of serum IL-6 to cross the blood-brain barrier (BBB). CAR-T cell therapy releases cytokines during endothelial cell (EC) stimulation, suggesting that the patient’s factors is as essential as those factors of CAR-T infusion for the associated toxicity. As the first line of defense against inflammatory stress, EC dysfunction and activity can affect disease severity and progression. The elevated biomarkers associated with endothelial activation, hemodynamic instability, capillary leakage, and coagulopathy observed in severe CRS further corroborated that CRS and ICANS may be mediated by endothelial activation and malfunction to a certain extent [13–18].

In this review, we aimed to highlight the role ECs play in intervening CAR-T cell-related toxicity and promoting the broader application of CAR-T cell therapy.

## Cytokine release syndrome and immune effector cell-associated neurotoxic syndrome

Studies using autologous anti-CD19 CAR-T cells have shown that CRS occurs in 42%–93% of patients and ICANS in 30%–67% [3, 19]. CRS is a systemic inflammatory response caused by CAR-T cell activation and proliferation and significant concomitant elevations of multiple serum cytokines [20], with the most pronounced peaks being IL-6, IL-1, interferon (IFN)-γ, and tumor necrosis factor-α (TNF-α), followed by IL-8, IL-10, monocyte chemotactic protein-1, and granulocyte-macrophage colony-stimulating factor [18, 21–23], leading to capillary leakage, elevated transaminases, and coagulation disorders [24–26]. Some of the clinical manifestations include fever, malaise, hypotension, shock, multiorgan dysfunction, and death [27, 28]. It can also result in severe CRS (grade ≥ 3) in up to 46% of patients [27–29].

**Fig. 1:**
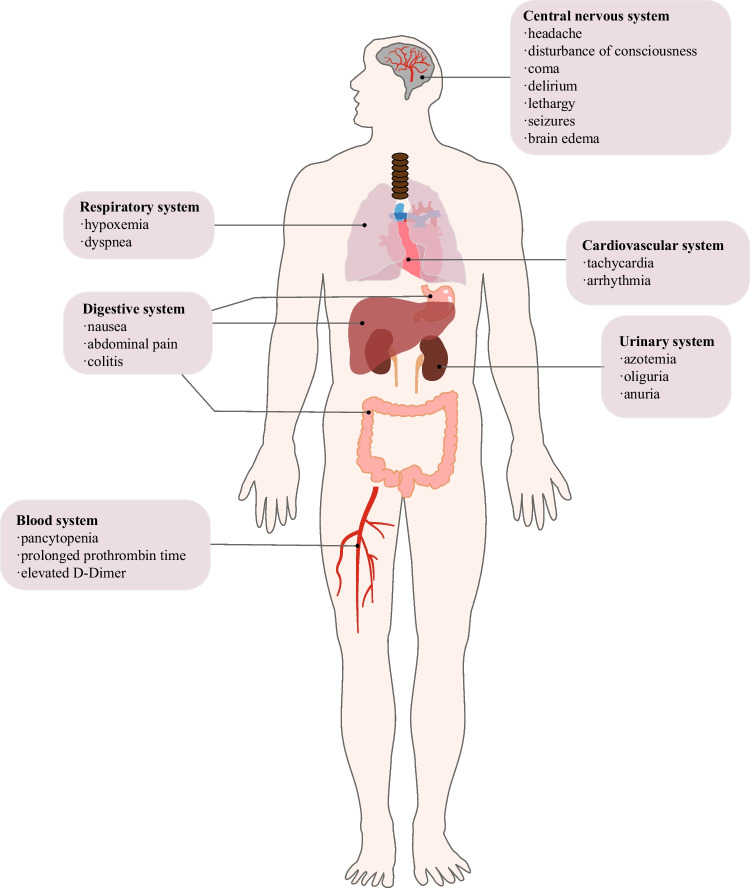
The clinical manifestations of CRS and ICANS

CAR-T cell therapy–associated neurotoxicity, on the other hand, is due to high levels of systemic inflammatory cytokines (IL-6, TNF-γ, and TNF-β), leading to EC activation, BBB destruction, and infiltration of peripheral cytokines and immune cells into the central nervous system (CNS) [15, 16, 27, 29–35], which subsequently initiates a feedback loop that continuously activates the endothelium, making toxic events irreversible. The most distinct risk factors for ICANS include systemic cytokine release and CRS severity [36]. The American Society for Transplantation and Cellular Therapy Consensus Panel on Toxicity [37] named this novel neurological syndrome “immune effector cell-associated neurotoxic syndrome,” and it can occur after or alone with CRS or can develop concurrently with CRS [28] and manifests as delirium, somnolence, coma, cognitive impairment, dysphagia, tremor, ataxia, myoclonus, sensory deficits, seizures, and cerebral edema [26] (Fig. 1).

## Biological behaviors of endothelial activation

Vascular ECs are essential for the activation of the coagulation system and the maintenance of vascular homeostasis. Healthy ECs naturally express substances that cause vasodilation, improve blood flow, decrease platelet (PLT) aggregation, and enhance fibrinolysis, whereas dysfunctional ECs lead to vasoconstriction and thrombosis. EC activation results in sequential activation of intracellular angiopoietin (Ang), TIE-2, JAK-STAT, PI3K-AKT, NF-kappa B, and other pathways, leading to the initiation of self-DNA damage apoptosis and the large-scale release of cytokines, such as IL-6 and IFN-γ, to participate in the “cytokine storm” while significantly upregulating a variety of adhesion molecules; promoting lymphocyte adhesion and infiltration into the organ interior [38], recruiting macrophages, neutrophils, and NK cells and promoting their activation; and releasing inflammatory mediators to join the inflammatory response [39].

EC activation has two outcomes: adaptive changes, which may be beneficial, and tissue-specific expression of vascular and secretory factors, which can facilitate organ repair and regeneration [40]. By contrast, excessive activation can lead to 1) damaged barrier function, which could allow soluble effectors to pass (such as TNF-α) and cause edema, damaging the endothelium; 2) an increased risk of thrombosis, which could lead to vascular thrombotic events (such as ischemia or stroke), and increased shear effects, which may result in erythrocyte fragmentation, anemia, thrombocytopenia, and neurological dysfunction; 3) increased expression of cell adhesion molecules (such as endothelial intercellular adhesion molecule-1 [ICAM-1], selectins, and integrins), leading to leukocyte migration and vascular adhesion, thus facilitating an inflammatory response and further exacerbating edema; and 4) upregulation of proinflammatory mediators. The upregulation of proinflammatory mediators is triggered by NF-κB translocation and Toll-like receptors. Inflammatory mediators enhance the expression of procoagulant tissue factors while inhibiting the anticoagulant system and indirectly activating the renin-angiotensin system [15, 40, 41]. In conclusion, inflammatory mediators created during EC activation continuously boost the activation process, resulting in a positive feedback loop and cytokine storms.

## Role of EC in CRS and ICANS

ECs are the first line of defense against inflammatory stress and are central regulators of cytokine storms [42]. In capillary leak syndromes (such as acute respiratory distress syndrome and severe burns), alternative markers of endothelial injury, such as IL-6, IL-8, and fibrinogen activator inhibitor-1, are elevated [43], suggesting a link between inflammation and endothelial dysfunction. Many prevalent and serious infectious diseases can be distinguished based on EC activation caused by inflammation. The degree of EC activation and subsequent dysfunction affect the severity and progression of the disease [44] .

CRS and ICANS have similar characteristics to the endothelial injury syndrome that occurs after hematopoietic cell transplantation (HCT) [26]. Vascular endothelial activation due to high levels of inflammatory cytokines is believed to favor the development of CRS and ICANS after CAR-T treatment. Cytokines such as IL-1 and IL-6 inhibit the natural anticoagulant pathway [45] and lead to the activation of ECs’ MAPK/NF-κB pathway [46–48], producing and releasing procoagulant granules, such as Weibel-Palade vesicles, while the cytoskeleton of ECs is recombined [49] and tight junctions are lost [50]. Brain microvascular ECs are critical regulators of systemic inflammatory signaling into the CNS; brain ECs mediate central febrile responses; express IL-1β, IL-6, and TNF receptors; and locally produce cytokines that enhance pro-inflammatory responses, altering endothelial transporter protein function [16, 40]. Clinical evidence supporting strong endothelial activation and enhanced BBB permeability has been found in patients with CRS and ICANS, including inflammation, impaired coagulation, and enhanced vascular permeability [15, 16, 18, 26]. Correlations between EC activation biomarkers, the development and severity of CRS, and the degree of liver, renal, and hematopoietic dysfunction also confirm that endothelial activation is one of the mechanisms for CAR-T cell immune-mediated toxicity after CAR-T cell therapy [51, 52].

**Fig. 2:**
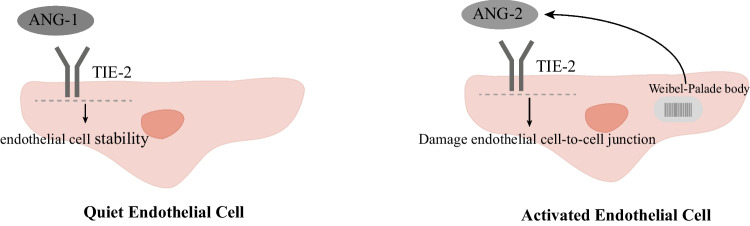
Simple schematic diagram of Ang-TIE-2 system. Ang, angiopoietin

The Ang-TIE-2 system may explain the association between endothelial activation, systemic cytokine release, and microvascular dysfunction [16]. TIE-2 is expressed on the EC surface [53], and Ang-1, produced mainly by perivascular cells and PLTs [16], binds to TIE-2 on the EC surface and activates downstream pathways to maintain EC stability. Ang-2, stored in endothelial Weibel-Palade vesicles, is released from EC upon EC activation, displacing Ang-1 and inhibiting TIE-2 signaling [54–57], thereby impairing EC-cell junctions, inducing expression of pro-inflammatory adhesion molecules (including ICAM-1 and vascular cell adhesion molecule-1 [VCAM-1]) and epithelial procoagulant protein levels [58–61]. The combination of high serum cytokine levels and endothelial activation leads to a cascade response that progressively amplifies endothelial activation and injury. Biomarkers of endothelial activation, such as Ang-2, Ang-2/Ang-1 ratio, and von Willebrand factor, are elevated in patients with severe CRS and severe neurotoxicity (grades 3–4) [16, 18, 30], and the Ang-2/Ang-1 ratio is elevated before lymphatic depletion in patients with grade 4 and 5 ICANS [16]. Patients with endothelial activation before CAR-T cell infusion are more prone to develop CRS and ICANS [18]. Furthermore, as PLTs produce Ang-1, changes in the Ang-2/Ang-1 ratio may be driven by thrombocytopenia alone, and patients with severe thrombocytopenia before or immediately following CAR-T cell infusion may be more susceptible to developing CRS and ICANS-related endothelial activation and damage. This is because PLTs are one of the limited sources of endothelium-stabilizing cytokines (Ang-1) [54] (Fig. 2).

EC activation is closely associated with immune-mediated inflammatory responses and organ damage; however, because the degree of EC activation varies among states, endothelial activation, a necessary step after initiation, is of great importance in intervening or mitigating the degree of endothelial activation in response to toxicity associated with CAR-T cell therapy.

## How to predict CRS and ICANS by endothelial related indexes

Lactate dehydrogenase (LDH), a surrogate marker of tumor infiltration, and the general inflammatory markers C-reactive protein (CRP) and ferritin all shared a connection with severe CRS and/or ICANS [18, 19, 62–66]. They could potentially be applied to predict CRS and ICANS risks in patients; however, the reliability of a single index is low. The endothelial activation and stress index (EASIX) score (LDH [U/L] × creatinine [mg/dL]/PLTs [10^9^ cells/L]) is a surrogate indicator of systemic endothelial activation that has been validated in the allogeneic hematopoietic stem cell transplantation [51, 67–73]; however, it is also applicable to other complex endothelial activation or injury situations, such as coronavirus disease 2019 [67–74]. It is significantly correlated with various endothelial dysfunction and complement activation biomarkers [75]. Higher EASIX score before CAR-T cell infusion parallels a significant elevation of multiple endothelial serum markers [17]. A study demonstrated that EASIX correlates with the incidence rate and seriousness of CRS and ICANS in axi-cel-treated large B-cell lymphoma patients [51]. This suggested that EASIX is also a useful prognostic marker of endothelial dysfunction in CAR-T cell therapy, with lateral evidence of EC involvement in the pathogenesis of CRS/ICANS. Modification of EASIX to remove creatinine (s-EASIX) or to replace creatinine with CRP (m-EASIX) was found to improve the predictive power [76, 77], and combining EASIX with inflammatory markers (CRP and ferritin) enhanced the prediction effect [51].

## Intervention strategies targeting the endothelium

**Fig. 3:**
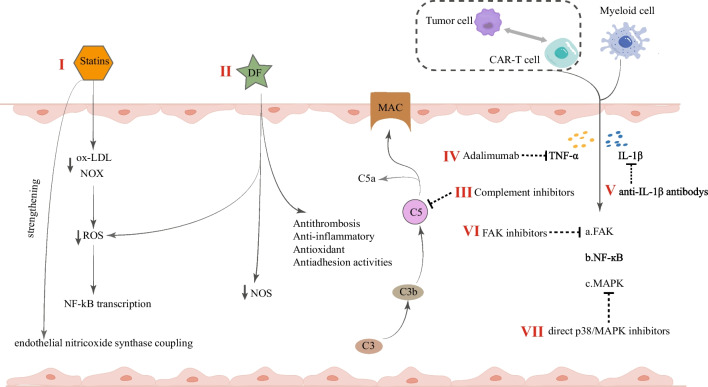
Intervention strategies to reduce endothelial activation and endothelial injury. ox-LDL, oxidized low-density lipoprotein; NOX, nicotinamide adenine dinucleotide phosphate oxidase; ROS, reactive oxygen species; DF, defibrillated polynucleotides; NOS, nitric oxide synthase; MAC, membrane attack complex; CART, chimeric antigen receptor (CAR)-T cell ; TNF-α, tumor necrosis factor-α; FAK, focal adhesion kinase

Intervention strategies to reduce endothelial activation and endothelial injury include statins, defibrillated polynucleotides, angiotensin-converting enzyme (ACE) inhibitors, angiotensin II receptor blockers, and a new type of p38/MAPK inhibitors [78] (Fig. 3).

### Statins

Statins regulate immunological responses at various levels, including immune cell adhesion and migration, antigen presentation, and cytokine production [79], and exert endothelial protection against inflammation and oxidative stress. Antagonizing Ang2 through the angiopoietin/Tie-2 signaling axis [80] exerts its endothelial protective function and maintains endothelial cell quiescence, which antagonizes the pathological process characteristic of endothelial dysfunction associated with hematopoietic stem cell transplantation and CAR-T cell therapy [81], and there is a clinical trial investigating the use of simvastatin and intrathecal dexamethasone for the prevention of neurotoxicity after treatment with axicabtagene-ciloleucel (NCT04514029) [81]. In addition, statins may help reduce oxidized low-density lipoprotein levels and nicotinamide adenine dinucleotide phosphate oxidase activity, thereby decreasing reactive oxygen species (ROS), impacting either directly or indirectly NF-κB transcription, or strengthening endothelial nitric oxide synthase coupling [41]; statins should be studied further as potential targets for the prevention and therapy of endothelial damage.

### Defibrillated polynucleotides

Defibrillated polynucleotides (DF) is a unique, naturally derived EC protector with potential effects on angiogenesis, EC activation, and endothelial inflammation, reducing EC activation through antithrombosis, anti-inflammatory, antioxidant, and antiadhesion activities [82], and restoring thrombolysis-fibrinolysis balance, with broad potential in a range of serious diseases based on EC injury and inflammation. The reduced release of inflammatory mediators manifests the anti-inflammatory effects, reduced production of ROS and nitric oxide synthase levels during oxidative stress [83, 84], and significant inhibition of heparanase expression and activity [85], promoting repair of endothelial integrity and function. DF significantly improved survival in patients with hepatic sinusoidal obstruction syndrome (SOS) and small hepatic vein occlusive disease (VOD) by stabilizing the endothelium [86, 87]. It has also been authorized to treat VOD/SOS in both adults and kids [88, 89], demonstrating effectiveness as well as safety. It is well tolerated and is reasonably presumed to be used to prevent or treat CAR-T cell immune-related toxicity. An in vitro study using endothelial cell lines revealed that defibrillating peptides inhibited endothelial proliferation in a dose-dependent manner and suppressed serum-induced endothelial activation and angiogenesis in graft-versus-host disease (GVHD) patients, and that DF had a significant positive effect on endothelial biological properties during aGVHD [83]. Another study found that adding defibrillating peptides reduced the alterations associated with endothelial dysfunction [90]. The administration of DF regulates EC injury in models of acute respiratory distress syndrome and idiopathic pneumonia syndrome [91]. A clinical trial showed that DF therapy slightly reduced the rate of CAR-T-associated neurotoxicity/high-grade event duration compared to previous data [92]. More research is required in the future to validate its efficacy.

### Complement inhibitors

The complement system is linked to EC activation, inflammation, leukocyte recruitment, PLT activation, and coagulation. All three pathways (classical, alternative, and lectin pathways) trigger proximal complement activation, leading to C3 activation and C3 convertase formation. Activation of C3 via the alternative complement route also amplifies this effect, ultimately leading to the deposition of C3 fragments on target cells. When sufficient C3b is deposited, the terminal cleavage pathway is triggered, leading to the formation of a membrane attack complex (MAC) on the surface of the target cells [26]. CAR-T cell-related toxicity can also lead to these changes, and endothelial damage continues to occur through the activation of a variety of complement pathways and interactions between complements and interferons.

Complement inhibition is safe and effective in patients with endothelial dysfunction syndromes (such as transplant-associated thrombotic microangiopathy) [26]. Complement inhibitors are currently approved by the Food and Drug Administration for the treatment of paroxysmal nocturnal hemoglobinuria, blocking terminal complement activation by binding to C5 so that the process of producing pro-inflammatory C5a molecules and MAC scleral complex formation is stopped. Complement inhibitors at the C3 and C5 levels reduced whole blood-induced endothelial cell activation by 89% and eliminated TNF release [93]; complement inhibition may be a viable new strategy to control the systemic complement-mediated inflammatory response.

### Adalimumab in combination with anti-IL-1β antibodies

During CAR-T cell treatment, vascular ECs are exposed to several stimuli in the bloodstream. Central cytokines that cause endothelial activation include IL-1β, which is released by activated myeloid cells, and TNF-α, which is produced by CAR-T cells when they recognize tumors. These cytokines highly activate EC by upregulating the expression of adhesion molecules (E-selectin, VCAM-1, and ICAM-1), while focal adhesion kinase (FAK) process, NF-кB process, and MAPK process are activated.

TNF receptor 1 (TNFR1), the primary TNF-α receptor on the endothelial membrane, is involved in the inflammatory process. By deleting TNFR1 in human ECs, the degree of CAR-T cell-induced endothelial activation was reduced. Endothelial activation was also prevented by selective small-molecule inhibitors of the TNFR1, NF-кB, and MAPK signaling pathways [94]. Adalimumab, anti-IL-1β, and FAK inhibitors effectively blocked TNF-α, IL-1β, and reduced FAK activity; improved endothelial dysfunction caused by CAR-T cells, malignant cells, and myeloid cells in CAR-T cells treatment; and reduced endothelial leakage caused by CAR-T and other cells. Moreover, the combination of adalimumab and anti-IL-1β antibodies showed synergistic effects [94]. As a result, the above drugs may have therapeutic potential for immunotoxicity associated with CAR-T therapy.

### Others

Corticosteroids are frequently used to regulate proinflammatory states linked to endothelium-related HCT complications, such as interstitial pneumonia syndrome, because they have anti-inflammatory properties that can reduce endothelial damage. However, they are only anti-inflammatory and not endothelial-specific. In order to combat endothelial activation and coagulation impairment, other strategies including increasing Ang-1 or PLT transfusions can be included [16, 55]. Symptomatic treatment with ACE inhibitors, angiotensin receptor blockers (ARBs), and cytokine inhibitors, such as IL-6 receptor antibodies, may be used for the different steps of the cascade response. ACE inhibitors [95, 96] and ARBs [97] are important strategies with endothelial protective potential because they have anti-inflammatory effects and improve endothelial dysfunction. As antagonists of the Ang-2 pathway, ACE inhibitors and ARB inhibit the p38/MAPK activation signal cascade reaction of endothelial cells, which is triggered by a high level of Ang-2 [78]; therefore, direct p38/MAPK inhibitors [98] might provide immediate and long-term protection of the endothelium by affecting the Ang-2 and p38/MAPK signaling axes, thus further improving patient outcomes.

## Conclusions and future directions

CAR-T cell therapy has achieved excellent results in patients with hematological malignancies. Its use has also been extended to other fields such as solid tumors; however, its response varies significantly among patients after infusion. ECs, an essential link in the role of CAR-T cells, are expected to be a target for intervening in CAR-T cell-related toxicity and promoting the broader application of CAR-T cell therapy. In the future, the relationship between EC dysfunction and the efficacy of CAR T-cell therapy should be investigated further.
